# Radiotherapy for Vaginal Recurrences of Cervical Cancer in Patients After Prior Surgery: Analysis of Effect and Prognostic Factors

**DOI:** 10.3389/fonc.2021.744871

**Published:** 2021-09-13

**Authors:** Junfang Yan, Ziye Zheng, Jiawei Zhu, Ke Hu, Xiaorong Hou, Jie Shen, Xin Lian, Shuai Sun, Zheng Miao, Jing Shen, Hui Guan, Qingyu Meng, Fuquan Zhang

**Affiliations:** Department of Radiation Oncology, Peking Union Medical College Hospital, Chinese Academy of Medical Sciences & Peking Union Medical College, Beijing, China

**Keywords:** vaginal recurrence, cervical cancer, radiotherapy, salvage radiotherapy, prognostic factor

## Abstract

**Objective:**

The role of salvage radiotherapy (RT) in the treatment for vaginal recurrence of cervical cancer in patients after prior surgery remains controversial. The aim of this study was to evaluate the efficacy and toxicity of salvage RT and explore prognostic factors associated with the survival after recurrence.

**Methods:**

Patients with cervical cancer, treated for vaginal recurrences at Peking Union Medical College Hospital between July 2011 and November 2019, were identified. All the patients underwent prior surgery for primary tumor and received salvage RT including external beam radiotherapy (EBRT), brachytherapy (BT), or both. The irradiation field and dose depended on the conditions of patients. Recurrence patterns were classified into four categories according to the site of recurrence. Prognostic factors on the overall survival (OS), progression-free survival (PFS), and local control (LC) were analyzed, and late toxicity was evaluated.

**Results:**

A total of 141 patients were included in the analysis, with a median follow-up time of 40.8 months. The estimated 5-year OS, PFS, and LC rates were 81%, 75%, and 87%, respectively. In multivariate analysis, endovaginal recurrence and no irradiation history were favorable prognostic factors associated with OS (all p < 0.05), PFS (all p < 0.05), and LC (all p < 0.05). The area under the receiver operating characteristic (ROC) curve (AUC) of the recurrence pattern is larger than the stage of primary tumor (0.734 *vs*. 0.670).

**Conclusions:**

RT was an effective treatment with tolerable toxicity for vaginal recurrences of cervical cancer in patients with prior surgery. Recurrence pattern and irradiation history were important prognostic factors.

## Introduction

Cervical cancer is the fourth most common cancer among females, which threatens the health of middle-aged and elderly women. Hysterectomy is an important part of surgery for early-stage cervical cancer (1, 2). Whether it is open surgery or minimally invasive surgery, 10%–30% cervical cancer patients experience disease relapse (3, 4). Vagina is the most common and important site of recurrence in cervical cancer (5).

For patients with vaginal recurrences, there is no consistent standard treatment (6). The optimal salvage treatment regimen includes salvage radiotherapy (RT), chemotherapy, and reoperation such as pelvic exenteration depending on the primary treatment, type of recurrence, and associated comorbidities. RT with or without concurrent chemotherapy as salvage treatment seems to present promising treatment outcomes and tolerable toxicities (7–9). Several studies have proved that salvage RT can achieve better survival outcomes (8, 10), especially when using brachytherapy (BT) for re-irradiation due to its conformal dose distribution (11, 12). However, studies on the effect and survival of RT as a salvage treatment for recurrent cervical cancer patients with prior surgery are still limited (13, 14). What is more, few studies paid attention to the effect of combined external beam radiotherapy (EBRT) and BT as salvage treatment, as well as prognostic factors associated with survival after vaginal recurrence.

In the present study, we collected data of patients with recurrent cervical cancer after hysterectomy surgery treated at Peking Union Medical College Hospital (PUMCH). All the patients experienced vaginal recurrence and received salvage RT. The first aim of this study was to evaluate the effect of salvage RT for vaginal recurrence. The second aim was to analyze the prognostic factors including characteristics, clinical factors, and treatment. Our institution has treated more than 200 patients with vaginal recurrences since 2011, and this study includes the largest sample size so far in this area.

## Material and Methods

### Patient Enrollment

One hundred and eighty female patients with vaginal recurrent cervical cancer, treated with salvage RT at Peking Union Medical College Hospital (PUMCH) between July 2011 and November 2019, were reviewed. Our inclusion criteria were listed as following: (1) underwent total hysterectomy surgery because of the primary tumor; (2) histologically confirmed that both the primary cancer and the recurrent tumor were cervical cancer, including squamous carcinoma and adenocarcinoma; (3) experienced vaginal recurrence and received EBRT, BT or both. The following exclusion criteria were adopted: (1) underwent hysterectomy because of benign disease; (2) refused to receive RT after recurrence; (3) experienced distant metastasis at the time of recurrence without vaginal recurrence; and (4) insufficient data of recurrence and salvage treatment. Finally, data of 141 patients met the criteria and were included. Initial stages of tumors at diagnosis were reclassified according to the 2018 International Federation of Gynecology and Obstetrics (FIGO) staging system for cervical cancer. Written informed consent was obtained from each patient.

### Treatment

All the patients received salvage RT for the vaginal recurrences by EBRT, BT, or both. The choice of RT technique, range of the irradiation field, and dose depended on the tumor size and site at the time of recurrence, as well as RT history. Among these patients, a combination of EBRT and BT was delivered to 103 patients; EBRT alone was delivered to 22 patients. The remaining 16 patients with recurrent tumors smaller than 5 mm, which was confined to the vaginal mucosa, received only BT, 5 Gy per fraction for four to six fractions.

As for patients with prior RT, most re-irradiation fields covered the recurrence regions and drainage fields of the involved lymph rather than the whole pelvic, while the prophylactic irradiation of the regional lymph node was rare. When the recurrent tumors were larger than 5 mm without prior RT, the irradiation field usually included the recurrence regions, upper 1/2 vagina, and paravaginal and pelvic lymphatic drainage areas. The whole vaginal area and bilateral inguinal lymph node drainage areas were added on this basis for patients with lower 1/3 vaginal segment relapse.

All the patients receiving EBRT were treated by VMAT or TOMO, with a gross tumor volume (GTV) dose of 30–80 Gy in 10–36 fractions. In patients who received BT, applicators were mainly multichannel vaginal cylinders, among which 21 patients adopted 3D-printed individual vaginal applicators. The BT dose was usually 5 Gy (range, 3–6 Gy) per fraction, mostly two to six fractions. When calculating the total dose of EBRT and BT, the biologically equivalent doses in 2-Gy fractions (EQD2) were utilized. The total EQD2 is the sum of EBRT and BT. The dose and fractionation schedules of EQD2 for EBRT were based on the GTV of tumor.

Before salvage RT, 14 patients had received other treatments for the recurrences, and 10 patients had undergone surgery (eight with vaginal tumorectomy and two with cytoreductive surgery) while 4 patients had adopted chemotherapy. Concurrent chemoradiotherapy was adopted for 73 patients.

### Follow-Up and Statistics

Failure patterns were divided to local recurrence, distant metastasis, and death. Late toxicity, defined as toxicity occurring more than 90 days after RT, was also evaluated. Most common late toxicities for EBRT and BT, such as toxicity of lower gastrointestinal and urinary tract, were assessed according to Common Terminology Criteria for Adverse Events (CTCAE) v3.0.

The primary endpoint was overall survival (OS), and the secondary endpoints were progression-free survival (PFS), local control (LC) rates, and late complications. OS was defined as the time interval between the date of recurrence and the date of death from any cause or the last follow-up. PFS and LC were calculated from the date of starting salvage RT to any recurrence, disease progression, or death. The Kaplan–Meier method was used to estimate OS, PFS, and LC rates. Log-rank tests and Cox proportional hazard regression methods were used to identify prognostic factors. Receiver operative characteristic (ROC) curves for OS and the values of area under the ROC curve (AUC) were created according to the recurrence pattern and tumor stage at diagnose. p values <0.05 were considered statistically significant. All analyses were performed using SPSS 23.0 (IBM Corp, Armonk, NY, USA).

## Results

### Patient and Treatment Characteristics

**Figure 1:**
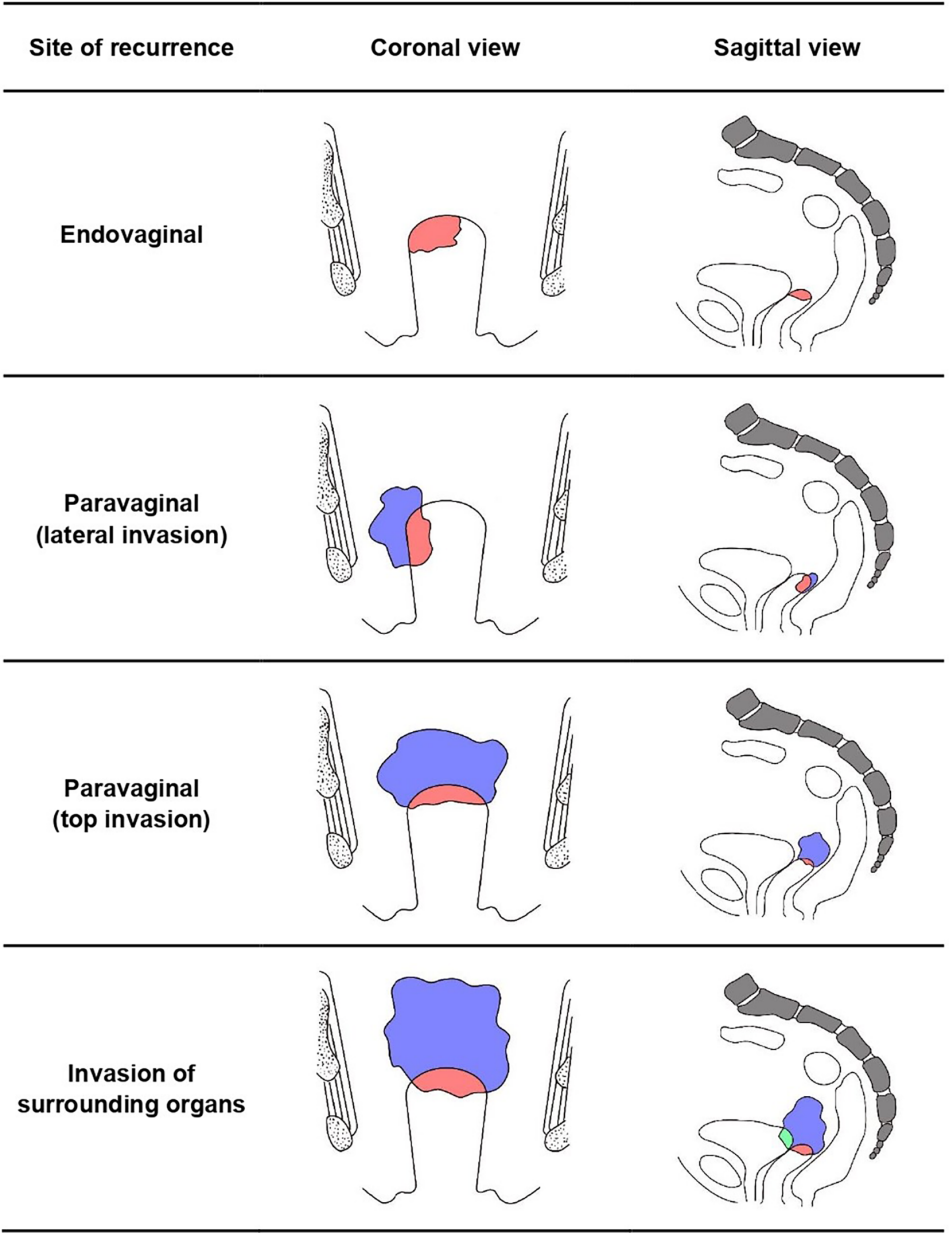
Schematic diagram of recurrence patterns.

A total of 141 patients were included in this study with a median age of 52 years, whose detailed characteristics and treatments are shown in **Table 1**. Recurrence patterns were classified into four categories according to the site of recurrence including: (1) endovaginal recurrence (n = 95); (2) paravaginal recurrence (n = 28), which means that the tumor invades paravaginal tissues or develops from the top of the vagina to the pelvic cavity, resulting in the limitation of vaginal movement; (3) recurrence invading surrounding organs (n = 17), such as the bladder, rectum, and pelvic wall; and (4) vaginal recurrence with distant metastasis (n = 1) (**Figure 1**).

**Table 1:** Patients and treatment characteristics.

Characteristics	Number	Percentage
Age, years, median (range)	52 (31–78)	
Histology		
Squamous carcinoma	133	94.3
Adenocarcinoma	8	5.7
Stage at diagnose (2018 FIGO staging system)		
I		
IA		
IA1	53	37.6
IA2	5	3.5
IB		
IB1	27	19.2
IB2	11	7.8
IB3	9	6.4
II		
IIA		
IIA1	13	9.2
IIA2	8	5.7
IIB	3	2.1
III		
IIIC		
IIIC1	11	7.8
IIIC2	1	0.7
Tumor size		
≤4 cm	121	85.8
>4 cm	20	14.2
Recurrence pattern		
Endovaginal	95	67.4
Paravaginal (include the top of vagina)	28	19.9
Invasion of surrounding organs (such as bladder, rectum, pelvic wall)	17	12.1
Distant metastasis	1	0.7
Lymph nodes metastasis		
Yes	48	34.0
No	93	66.0
RT dose (EQD2, Gy), median (range)	64.9 (25.0–95.8)	
RT treatment		
EBRT+BT	103	73.0
EBRT	22	15.6
BT	16	11.4
Re-irradiation		
Yes	32	22.7
No	109	77.3
BT technique		
2D	88	62.4
3D	31	22.0
No	22	15.6
Treatment for recurrence before RT		
Yes	14	9.9
No	127	90.1
Concurrent chemoradiotherapy		
Yes	73	51.8
No	68	48.2

FIGO, International Federation of Gynecology and Obstetrics; RT, radiotherapy; EBRT, external beam radiotherapy; BT, brachytherapy; EQD2, equivalent dose of 2 Gy per fraction.

The median interval between primary treatment and tumor relapse was 24.0 months (range, 2.5 to 238.0 months). The median EQD2 of the salvage RT was 64.9 Gy (range, 25.0 to 95.8 Gy). For the 16 patients that received BT only, the EQD2 ranged from 25.0 Gy to 37.5 Gy. Re-irradiation was performed in 32 patients with a median EQD2 of 62.9 Gy (range of 31.3-95.8 Gy). Besides, the other 98 patients received RT with a median EQD2 of 69.2 Gy (range, 45.0 Gy to 93.4 Gy).

### Clinical Outcomes

**Figure 2:**
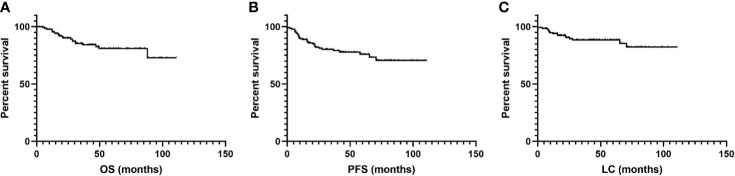
Kaplan–Meier curves for **(A)** overall survival (OS), **(B)** progression-free survival (PFS), and **(C)** local control (LC) after salvage radiotherapy treatment for recurrence.

The median follow-up time was 40.8 months (range, 2.0 to 110.9 months) with an estimated 3-year OS of 85%. The estimated 5-year OS, PFS, and LC rates were 81%, 75%, and 87%, respectively (**Figure 2**). Disease progression during follow-up was confirmed in 32 patients, among which 11 patients experience local recurrence, 17 patients experience distant metastasis, and 4 patients experience both.

### Prognostic Factors Associated With OS, PFS, and LC

Factors including histological type (squamous carcinoma or adenocarcinoma), tumor size (≤4 *vs*. >4 cm), tumor stage at diagnosis (classified by the 2018 FIGO stage system), recurrence pattern (endovaginal, paravaginal, invasion of surrounding organs or distant metastasis), lymph node metastasis, history of radiotherapy (re-irradiation, with or without), time from initial treatment to recurrence (≤7 *vs*. >7 months), RT dose (EQD2 ≤ 40 Gy, 40–65 Gy, or > 65 Gy), BT technique (2D, 3D, or no BT), treatment for recurrence before RT, and use of concurrent chemoradiotherapy were analyzed, as shown in **Table 2**.

**Table 2:** Factors predictive of overall survival (OS), progression-free survival (PFS), and local control (LC).

Variables	Median OS (mo)	*P* value		Median PFS (mo)	*P* value		Median LC (mo)	*P* value	
		Univariate (OS)	Multivariate (OS)		Univariate (PFS)	Multivariate (PFS)		Univariate (LC)	Multivariate (LC)
Histology									
Squamous carcinoma	40.77	0.857		36.99	0.583		40.08	0.313	
Adenocarcinoma	43.17			43.17			35.25		
Tumor size									
≤4 cm	41.73	0.002		40.77	<0.001		40.77	0.060	
>4 cm	34.17		0.610	23.69		0.938	27.58		
Stage at diagnose (FIGO stage)									
IA1	45.31	<0.001	0.059	43.66	<0.001	0.362	44.16	0.071	
IA2	49.73		0.782	49.73		0.980	49.73		
IB1	32.23		0.198	30.72		0.562	30.72		
IB2	37.22		0.923	35.42		0.840	35.42		
IB3	37.52		0.178	37.52		0.062	37.52		
IIA1	42.55		0.912	42.55		0.779	42.55		
IIA2	26.25		0.123	25.00		0.571	25.00		
IIB	14.09		<0.001	7.56		0.009	7.56		
IIIC1	17.12		0.471	9.43		0.336	12.32		
IIIC2	14.36		0.379	9.232		0.831	14.36		
Recurrence pattern									
Endovaginal	45.24	<0.001	0.004	44.26	<0.001	<0.001	44.26	0.001	0.007
Paravaginal	30.90		<0.001	25.50		<0.001	26.60		0.001
Invasion of surrounding organs	25.69		0.012	16.13		0.011	18.17		0.004
Distant metastasis	8.54		0.999	8.54		0.996	8.54		0.988
Lymph nodes metastasis									
Yes	27.61	<0.001	0.181	21.63	<0.001	0.061	23.67	0.007	0.765
No	45.31			44.75			45.24		
Re-irradiation									
Yes	31.00	0.002	0.020	21.95	<0.001	0.002	21.95	<0.001	<0.001
No	43.53			42.32			42.55		
Time from initial treatment to recurrence									
≤7 months	31.87	0.035		29.29			26.27		
>7 months	41.92		0.304	40.77	0.213		41.73	0.773	
RT dose (EQD2, Gy)									
≤40	64.30	0.107		64.61	0.493		64.61	0.875	
40–65	41.96			38.05			38.05		
>65	35.19			31.01			32.00		
BT technique									
2D	53.21	0.081		51.04	0.111		53.21	0.079	
3D	25.53			21.91			24.81		
No BT	25.91			22.65			20.80		
Treatment for recurrence before RT									
Yes	50.28	0.342		50.28	0.335		50.28	0.423	
No	37.52			35.42			36.21		
Concurrent chemoradiotherapy									
Yes	31.01	0.027	0.094	25.50	0.124		26.94	0.463	
No	45.91			44.76			45.91		

FIGO, International Federation of Gynecology and Obstetrics; BT, brachytherapy.

**Figure 3:**
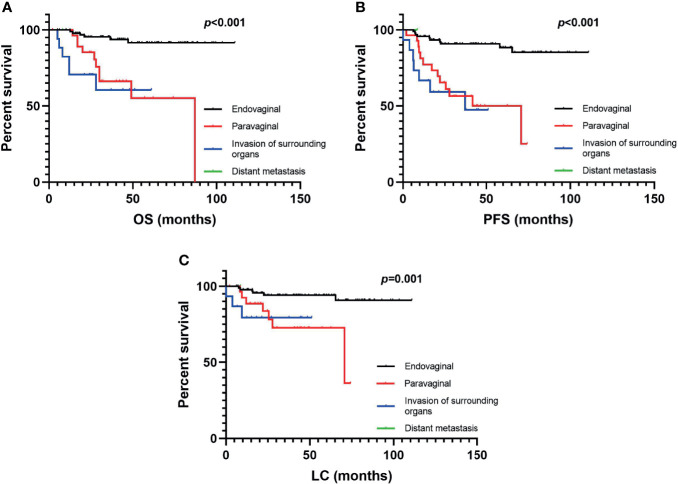
The **(A)** overall survival, **(B)** progression-free survival, and **(C)** local control rates of vaginally recurrent patients in different recurrence patterns.

In terms of OS, univariate analysis demonstrated that all the factors except for histological type and treatment for recurrence before RT were statistically significant. In multivariate analysis, recurrence pattern, history of radiotherapy, and stage at diagnosis were independent prognostic factors for OS (**Table 2**). Endovaginal recurrence (*p* < 0.05, **Figure 3**) was associated with prolonged OS, while invasion of surrounding organs (*p* < 0.05, **Figure 3**), prior RT history (*p* < 0.05), and IIB stage at diagnosis were negative prognostic factors of OS. Multivariate analysis also revealed that endovaginal recurrence and being RT naïve were independent factors for improved PFS (**Table 2**), while IIB stage at diagnosis was the negative prognostic factor for PFS. In terms of LC, recurrence pattern and re-irradiation were independent factors (**Table 2**).

**Figure 4:**
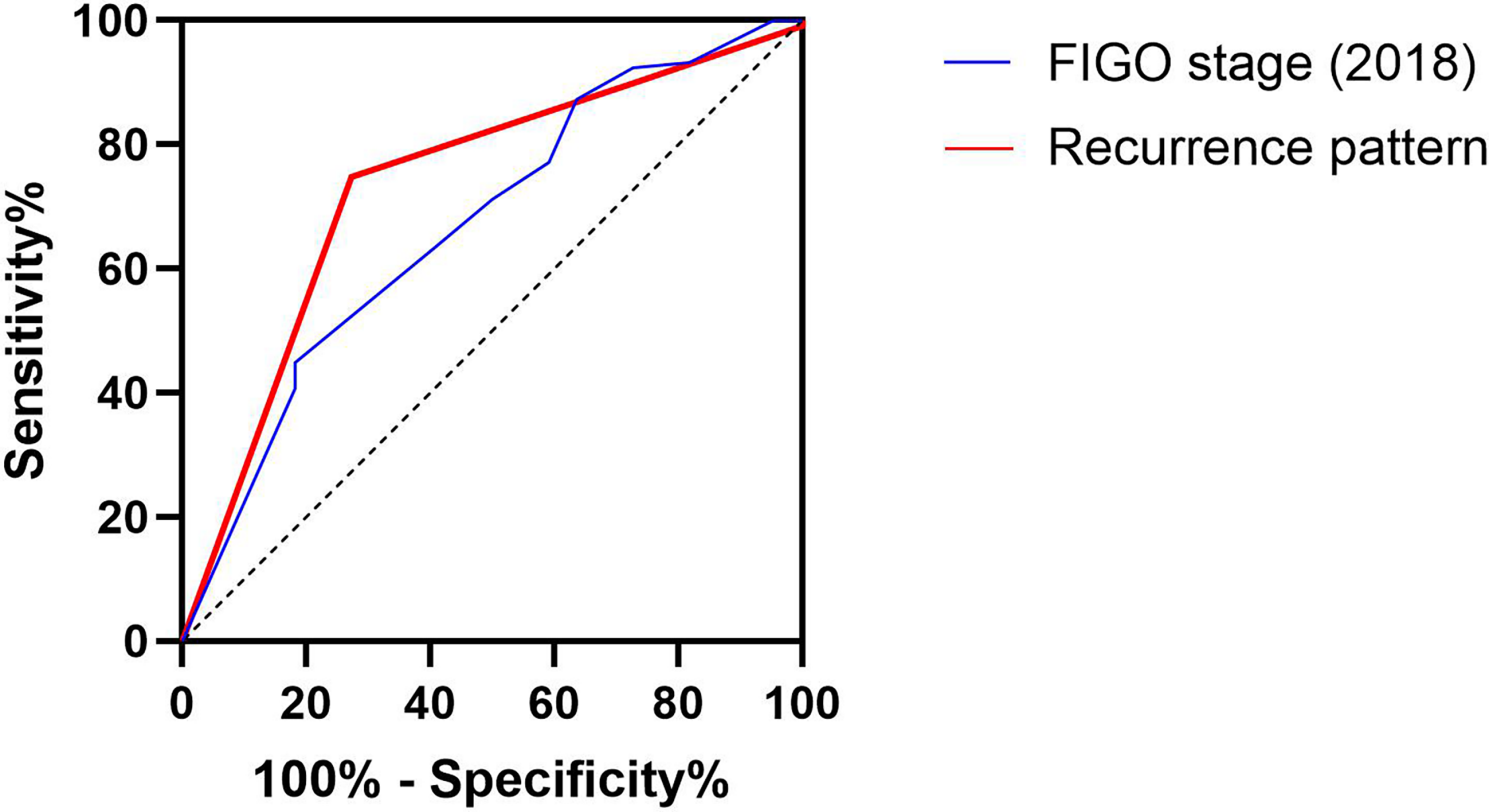
Receiver operative characteristic (ROC) curve of the predictive accuracy for OS of patients by FIGO stage (2018) and recurrence pattern.

The receiver operating characteristic (ROC) curve was utilized to compare the depictive accuracy of our recurrence pattern and initial stage of tumor at diagnosis on OS (**Figure 4**). The area under the ROC curve (AUC) value of the recurrence pattern was 0.734 (95% CI: 0.618 to 0.851, *p* < 0.001), while the AUC of the initial stage was 0.670 (95% CI: 0.545 to 0.794, *p* = 0.012).

### Toxicity and Late Complications

Twenty-two (15.6%) patients experienced grade ≥2 late complications. Grade ≥4 late complications were observed in six patients, and five of them were patients with re-irradiation (**Supplementary 1**). Five patients experienced rectovaginal fistula, and one patient experienced intestinal fistula. One patient suffered from vesicovaginal fistula.

## Discussion

The selection of treatment for vaginal recurrence of cervical cancer remained challenging and highly individualized. Surgical therapy was beneficial for patients with vaginal recurrence, particularly for patients without RT history (9). However, the quality of life might be strongly affected because of the shortened length of vagina (15). For local recurrence cervical cancer confined to the central pelvis, pelvic exenteration could be a potentially curable treatment, but the survival was still limited (16). Surgical mortality was nearly 5%, and the survival rate was less than 50% in carefully selected patients (17–19). Besides, the effects on patients’ physical, mental, and self-image features reduced the quality of life of patients (16, 20).

RT presented excellent effects and survival outcomes in recurrent cervical cancer patients with tolerable toxicity. Huang et al. reported a 2-year LC rate of 60% in recurrent cervical cancer patients treated with interstitial BT (21). There were a few studies focusing on the role of salvage RT in cervical cancer patients with postoperative recurrence. Kim et al. reported that the 5-year PFS and OS rates were 62.7% and 60.1%, respectively (22). Similar results were observed in another retrospective study with a 5-year OS of 66% and a 5-year local failure-free survival (LFFS) of 63.9% (23). The results of the present study demonstrated that RT was an effective salvage treatment for recurrent patients, with excellent 5-year OS, PFS, and LC rates of 81%, 75%, and 87%. Compared with the median EQD2 that ranged from 45 to 82.5 Gy in previous studies, the median EQD2 of the salvage RT was 64.9 Gy (range, 25.0 to 95.8 Gy) in this study (7, 8, 11, 12, 22), which indicated that larger RT doses contributed to the outstanding clinical outcomes. The application of advanced RT techniques, including volumetric modulated arc therapy (VMAT), TOMO, 3D-BT, intracavitary with or without interstitial (IC ± IS) technique, and individualized RT field design for patients with re-irradiation, all contributed to the delivery of higher RT doses in this study.

Factors associated with prognosis of salvage RT for recurrent cervical cancer were complicated. In previous studies, cervical squamous carcinoma, smaller recurrent lesion size, no lymph node metastasis, and no irradiation history tended to improve the OS (11, 14, 23). The most innovative finding of this study was that we defined the recurrence pattern according to the recurrence site and tumor invasion and found that it was strongly associated with the OS, PFS, and LC. The better survival of endovaginal recurrence could be attributed to the higher feasibility for performing BT, while the RT dose for extravaginal recurrence was limited by the surrounding organs, such as bladder, rectum, and intestine, making it difficult to perform radical RT and BT in these patients. Although some of these patients were given a higher dose through combined 3D-printed applicators or interstitial implantation BT, a second recurrence and distant metastasis were still more common than in those patients with endovaginal recurrence receiving a lower dose through simpler RT techniques. Several studies have consistently demonstrated pelvic wall involvement as an unfavorable prognostic factor (13, 14, 24). Even when receiving salvage RT with concurrent chemotherapy, the 5-year OS of patients with pelvic wall involvement was still below 30% (22).

The AUC value for the vaginal recurrence pattern was higher than the AUC value for the initial stage in this study (0.734 *vs*. 0.670). It revealed that the recurrence pattern according to the recurrence site and tumor invasion presented a more convincing predictive accuracy for OS. There was neither emphasis on adjustment of staging after recurrence in the National Comprehensive Cancer Network (NCCN) guideline for cervical cancer nor an independent recurrence stage in the FIGO staging system. The possible reason might be the relatively low ratio of recurrent cervical cancer patients after standard treatments. However, the recurrence site and the recurrence area needed to be taken into consideration once recurrence occurs. Our recurrence pattern could be a useful supplement for staging, selection of treatment and prediction of prognosis.

In our study, only initial stage IIB was significantly associated with unfavorable OS and PFS, and the differences were not presented in any other stages (**Table 2**). What is more, the initial stage of IIB tended to present a worse prognosis than IIIC (**Supplementary 2, Supplementary 3**), which was inconsistent with the staging we expected. According to the NCCN guideline for cervical cancer, the preferred initial treatment for IIB stage patients is RT with concurrent chemotherapy (18). Attempt at surgery might be one of the reasons for this unsatisfactory prognosis. Gupta et al. compared the efficacy of neoadjuvant chemotherapy followed by radical surgery *versus* chemoradiation in stage IIB cervical cancer patients and found that the 5-year disease-free survival (DFS) rate of the surgery group was 67.2%, significantly lower than 79.3% in the chemoradiation group (25). The diagnosis of the IIB stage mainly depended on physical examination, which was of high subjectivity, and the use of magnetic resonance imaging (MRI) might increase the accuracy of preoperative evaluation. What is more, most of these IIB stage patients had received RT during the initial treatment, so the poor OS and PFS may also attribute to re-irradiation.

Prior RT history was also one of the most important prognostic factors, which was consistent with the study by Kim (23). As for RT-naïve patients, higher RT doses could be delivered resulting in a better tumor remission. However, the dose of re-irradiation was limited because of the initial radiation damage on the surrounding tissue and the potential severe radiogenic side effects, especially for in-field recurrence (7). In a previous study, the 5-year LFFS in postoperative recurrent patients without RT history receiving salvage RT was 63.9%, while it was 47.1% in patients that received re-irradiation (23).

One principle of RT is to maximize the tumor dose while minimizing the dose of the neighboring tissue and organ at risk (26). High-dose or repeated RT could increase the risk of severe radiation toxicity. The rates of grade ≥2 lower urinary tract toxicity and lower gastrointestinal toxicity were 5.0% and 10.6% in our study, respectively. According to previous studies, grade ≥2 late complications of RT for recurrent patients are usually tolerable, with an approximate rate lower than 15% (21, 23, 27). It has been reported that re-irradiation might increase the risk of complication up to 15%–20% (12, 28). Compared with previous studies, our study presented an acceptable rate of grade ≥2 late complications (15.6%), which can also be attributed to the high RT dose in our treatment regimens.

The main limitation of this study was its retrospective, non-random design and inconsistency in treatment management due to the long-time span. The tumor characteristics of patients were quite different, and the time span of this study was relatively long, leading to the non-uniform radiotherapy technology and dose of patients. It was a pity that no optimal dose or RT plan for different vaginal recurrence patterns was summarized from this study. Besides, potential bias might be introduced during patient selection of the initial surgery treatment and recurrent treatment. Last but not least, because of the lack of standard guideline for the treatment of recurrent cervical cancer, selection of the treatment plans and RT techniques largely depended on the experiences of each medical center, which might lead to the heterogeneity. Future studies are needed to design standard treatment plans and evaluate the effect of RT in recurrent cervical cancer according to the recurrence pattern and recurrence sites.

In conclusion, for vaginal recurrences of cervical cancer in patients after prior surgery, salvage RT was an effective treatment with tolerable toxicity. Recurrence pattern based on the recurrence site and tumor invasion was a significant prognostic factor on OS, PFS, and LC. Endovaginal recurrence and no irradiation history were associated with better outcomes.

## Data Availability Statement

The original contributions presented in the study are included in the article/**Supplementary Material**. Further inquiries can be directed to the corresponding author.

## Author Contributions

JY and ZZ collected, analyzed, and interpreted the data and wrote the manuscript. JZ interpreted the data and wrote the manuscript. KH, XH, JieS, XL, SS, ZM, JingS, HG, and QM reviewed and edited the manuscript. FZ designed the study, interpreted the data, critically reviewed the manuscript, and supervised the study. FZ has full access to all the data in the study and final responsibility for the decision to submit for publication. All authors contributed to the article and approved the submitted version.

## Conflict of Interest

The authors declare that the research was conducted in the absence of any commercial or financial relationships that could be construed as a potential conflict of interest.

## Publisher’s Note

All claims expressed in this article are solely those of the authors and do not necessarily represent those of their affiliated organizations, or those of the publisher, the editors and the reviewers. Any product that may be evaluated in this article, or claim that may be made by its manufacturer, is not guaranteed or endorsed by the publisher.
