# Supplementary figures and images for: Radiotherapy for Vaginal Recurrences of Cervical Cancer in Patients After Prior Surgery: Analysis of Effect and Prognostic Factors
Source: Front Oncol. 2021 Sep 13;11:744871. doi: 10.3389/fonc.2021.744871 (PMC8475272; doi:10.3389/fonc.2021.744871)

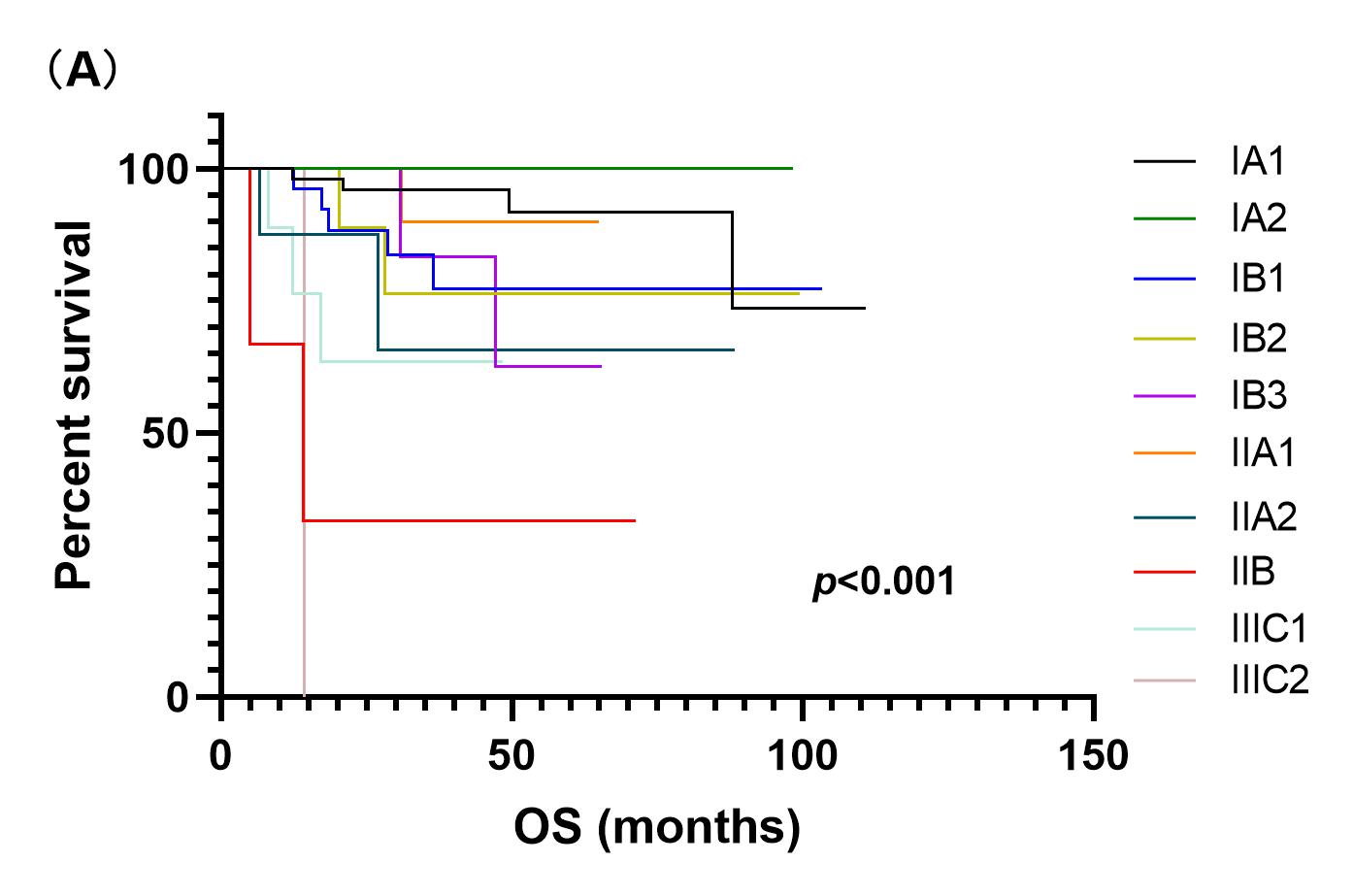

Supplement: Supplementary file 1 [file Image_1.jpeg]

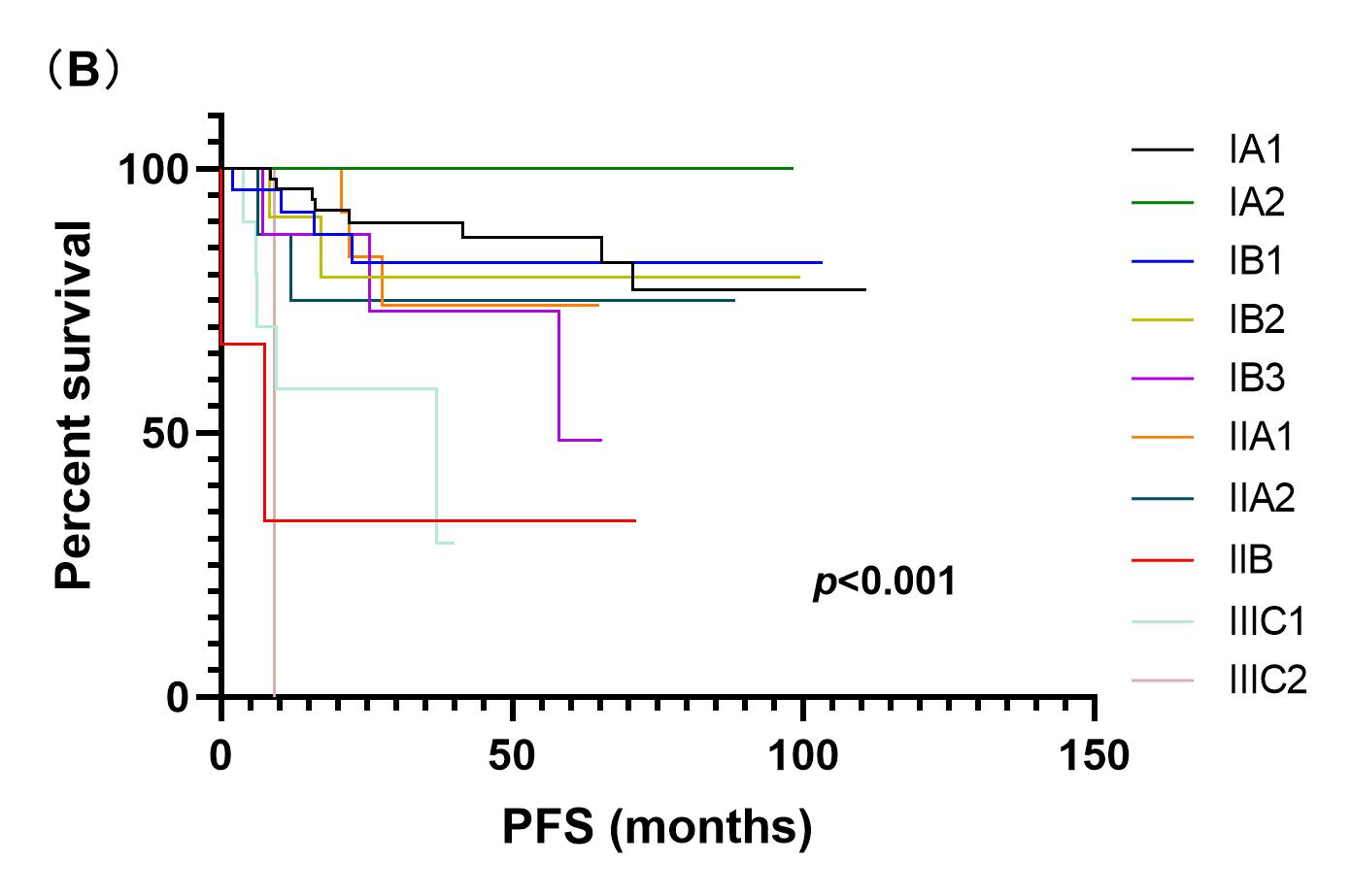

Supplement: Supplementary file 2 [file Image_2.jpeg]
