# Supplementary material for: Radiotherapy for Vaginal Recurrences of Cervical Cancer in Patients After Prior Surgery: Analysis of Effect and Prognostic Factors
Source: Front Oncol. 2021 Sep 13;11:744871. doi: 10.3389/fonc.2021.744871 (PMC8475272; doi:10.3389/fonc.2021.744871)
Supplement: Supplementary file 3 [file Table_1.docx]

**Supplementary 1. Late complications in RT naïve patients and patients with re-irradiation.**

| Late complication | RT naïve | Re-irradiation |
| --- | --- | --- |
| Lower gastrointestinal toxicity |  |  |
| Grade 2 | 5 | 2 |
| Grade 3 | 1 | 1 |
| Grade 4 | 1 | 5 |
| Urinary tract toxicity |  |  |
| Grade 2 | 6 | 0 |
| Grade 3 | 0 | 1 |
